# Association between Modifiable Risk Factors and Levels of Blood-Based Biomarkers of Alzheimer’s and Related Dementias in the Look AHEAD Cohort

**DOI:** 10.14283/jarlife.2024.1

**Published:** 2024-01-05

**Authors:** K.M. Hayden, M.M. Mielke, J.K. Evans, R. Neiberg, D. Molina-Henry, M. Culkin, S. Marcovina, K.C. Johnson, O.T. Carmichael, S.R. Rapp, B.C. Sachs, J. Ding, H. Shappell, L. Wagenknecht, J.A. Luchsinger, M.A. Espeland

**Affiliations:** 1 Department of Social Sciences and Health Policy, Wake Forest University School of Medicine, Winston-Salem, NC, USA; 2 Department of Epidemiology and Prevention, Wake Forest University School of Medicine, Winston-Salem, NC, USA; 3 Department of Biostatistics and Data Science, Wake Forest University School of Medicine, Winston-Salem, NC, USA; 4 Winston-Salem State University, Winston-Salem, NC, USA; 5 Alzheimer’s Therapeutic Research Institute, Keck School of Medicine, University of Southern California, San Diego, CA, USA; 6 Medpace Reference Laboratories, Cincinnati, OH, USA; 7 Department of Preventive Medicine, University of Tennessee Health Science Center, Memphis, TN, USA; 8 Biomedical Imaging Center, Pennington Biomedical Research Center, Baton Rouge, LA, USA; 9 Department of Psychiatry & Behavioral Medicine, Wake Forest University School of Medicine, Winston-Salem, NC, USA; 10 Department of Neurology, Wake Forest University School of Medicine, Winston-Salem, NC, USA; 11 Sticht Division of Gerontology and Geriatric Medicine Research, Wake Forest University School of Medicine, Winston-Salem, NC, USA; 12 Departments of Medicine and Epidemiology, Columbia University Irving Medical Center, New York, NY, USA

**Keywords:** Alzheimer’s disease, blood-based biomarkers of AD, comorbidities, obesity, diabetes

## Abstract

**Background:**

Emerging evidence suggests that a number of factors can influence blood-based biomarker levels for Alzheimer’s disease (AD) and Alzheimer’s related dementias (ADRD). We examined the associations that demographic and clinical characteristics have with AD/ADRD blood-based biomarker levels in an observational continuation of a clinical trial cohort of older individuals with type 2 diabetes and overweight or obesity.

**Methods:**

Participants aged 45-76 years were randomized to a 10-year Intensive Lifestyle Intervention (ILI) or a diabetes support and education (DSE) condition. Stored baseline and end of intervention (8-13 years later) plasma samples were analyzed with the Quanterix Simoa HD-X Analyzer. Changes in Aβ42, Aβ40, Aβ42/Aβ40, ptau181, neurofilament light chain (NfL), and glial fibrillary acidic protein (GFAP) were evaluated in relation to randomization status, demographic, and clinical characteristics.

**Results:**

In a sample of 779 participants from the Look AHEAD cohort, we found significant associations between blood-based biomarkers for AD/ADRD and 15 of 18 demographic (age, gender, race and ethnicity, education) and clinical characteristics (APOE, depression, alcohol use, smoking, body mass index, HbA1c, diabetes duration, diabetes treatment, estimated glomerular filtration rate, hypertension, and history of cardiovascular disease) .

**Conclusions:**

Blood-based biomarkers of AD/ADRD are influenced by common demographic and clinical characteristics. These factors should be considered carefully when interpreting these AD/ADRD blood biomarker values for clinical or research purposes.

## Introduction

Alzheimer’s disease (AD) blood-based biomarkers have evolved rapidly over the last decade with the development of more sensitive technologies, such as single molecular array (SIMOA) immunoassays, to more accurately measure biomarkers of AD pathology (Aβ42/Aβ40 ratio and phosphorylated tau at threonine 181 [ptau181]), and neurodegeneration (neurofilament light chain, [NfL] and glial fibrillary acidic protein [GFAP]) (1-3). However, with the increasing availability of blood biomarker assays for clinical use, there is an urgent need to understand their utility in large, heterogeneous populations. Several recent studies have reported that multiple chronic conditions can affect the interpretation of the blood biomarkers, causing increases or decreases in levels due to peripheral physiological factors such as obesity or renal function (4-8). Understanding the impact of multiple chronic conditions on AD biomarker levels in diverse populations will inform their use and interpretation in clinical settings.

The Action for Health in Diabetes (Look AHEAD) MIND cohort provides an opportunity for a unique contribution to this emerging body of literature in the high AD and Alzheimer’s related dementia (ADRD) risk group of persons with type 2 diabetes (T2D) (9). Look AHEAD participants represent a diverse group of individuals with T2D and overweight or obesity, who were recruited from 16 sites across the US who were randomized 1:1 to a lifestyle intervention vs. a control condition (10). To date there have been few, if any, studies that have comprehensively examined the AD/ADRD blood-based biomarkers in this type of cohort. Also, associations between diabetes duration and the AD/ADRD blood-based biomarkers are not known. Herein we present descriptive data on baseline levels of Aβ42, Aβ40, Aβ42/Aβ40, ptau181, NfL, and GFAP, and factors associated with their levels, as well as change over time from baseline to the end of the intervention.

## Methods

### Study design

**Figure 1.**
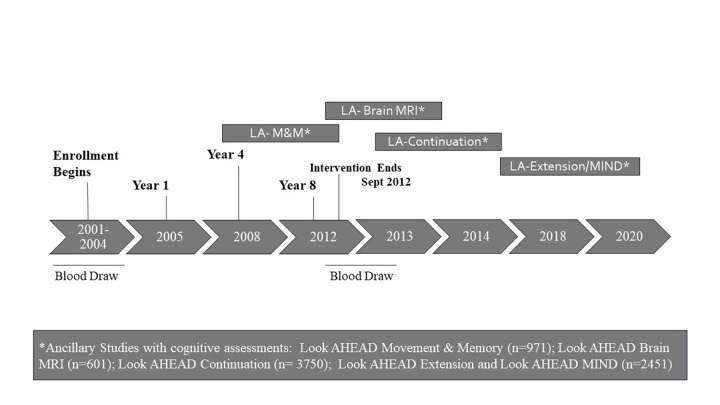
Look AHEAD Timeline

Look AHEAD was a randomized controlled clinical trial of participants with T2D and overweight or obesity. The study design, methods (10), and CONSORT diagram (11) for Look AHEAD have been published previously. The trial was designed to determine whether intentional weight loss for older adults with diabetes and overweight/obesity provided health benefits. The primary outcome of the trial was a composite of fatal and major not-fatal cardiovascular events. Eligibility criteria required that participants have body mass index (BMI) >25 kg/m^2^ (>27 kg/m^2^ if on insulin), glycated hemoglobin (HbA1c) <11%, systolic/diastolic blood pressure <160/100 mmHg, and triglycerides <600 mg/ dl. Participants were required to demonstrate over a two-week run-in period, the ability to make a daily record of their diet and physical activity. A behavioral psychologist or interventionist met with each participant to confirm that the intervention requirements were understood and that participants did not have competing life stressors that would impair adherence to the protocol. Enrollment spanned 2001 to 2004. The study was halted in September 2012 for futility with respect to the primary outcome and follow-up has continued as an observational study (see timeline, Figure 1). Local Institutional Review Boards approved the protocols and all participants provided written informed consent.

### Setting, Participants, and Intervention

Participants (n=5,145) aged 45-76 years were recruited from 16 sites across the United States and were randomized to either an Intensive Lifestyle Intervention (ILI) or a Diabetes Support and Education (DSE) condition. The ILI was a multidomain intervention including dietary modification and physical activity with a goal of inducing an average of ≥7% weight loss at one year and maintenance of weight loss over the course of the study (12). Participants in the ILI arm had a daily calorie goal of 1200 to 1800 kcal based on initial weight. The diet specified <30% total calories from fat (<10% saturated fat) and a minimum of 15% total calories from protein. The physical activity goal was similar in intensity to brisk walking for at least 175 minutes per week. The participants randomized to the DSE condition were invited, but not required, to attend three yearly group sessions. These sessions focused on diet, physical activity, and social support (13). There were no specific instructions or goals for weight loss, physical activity, or dietary modification. The current analyses include those who participated in blood draws at baseline and in 2010-2013 participated in cognitive interviews. Preference for inclusion was given to those who also participated in a brain MRI ancillary study. Compared to those included in the study, participants who were not included were younger, more were women, and more often self-identified as American Indian/Native American/Alaskan Native or Hispanic. Excluded participants had higher levels of education, higher baseline BMI, and fewer had a baseline history of cardiovascular disease (CVD), as seen in Supplemental Table 1.

### Biomarkers

Blood samples were drawn at baseline and proximal to the end of the intervention, approximately 8-13 years later. All assays were performed using EDTA plasma samples stored at Medpace Reference Laboratories, Cincinnati, OH. Concentrations of Aβ42, Aβ40, GFAP, and NfL were measured using the Simoa Human Neurology 4-Plex E Advantage Kit on a Quanterix Simoa HD-X bead-based immunoassay analyzer (Quanterix Corporation, Billerica, MA). The determination of the concentration of the pTau-181 was performed using the Simoa pTau-181 V2 Advantage kit. For all assays, within-run coefficients of variation (CVs) ranged from 3-19% and between-run CVs ranged from 6-13%. Biomarkers were converted to z-scores using baseline sample means and standard deviations for comparability.

### Baseline demographic and clinical characteristics

Demographic characteristics were collected at baseline and included age; self-identified gender, race, and ethnicity; years of education; alcohol use; and smoking status. Participants provided self-reports of clinical characteristics including the duration of their diabetes, type of diabetes treatment (diet, oral medications, oral medications and insulin), hypertension, high cholesterol, history of CVD (including MI, heart bypass surgery, coronary artery bypass graft, carotid endarterectomy, lower leg angioplasty, aortic aneurysm, congestive heart failure, or stroke), peripheral neuropathy, and use of antidepressants. Additional variables measured at baseline included BMI, systolic and diastolic blood pressure, and depressive symptoms (Beck Depression Inventory, BDI (14)). Blood specimens were analyzed centrally at the Northwest Lipid Metabolism and Diabetes Research Laboratories at the University of Washington for HbA1c and creatinine. Estimated glomerular filtration rate (eGFR) was calculated. APOE ε4 carrier status was determined for participants who provided consent (80% of women versus 86% of men, p<0.001), using TaqMan genotyping (rs7412 and rs429358) (15).

### Statistical Methods

Demographic and clinical characteristics of the sample were compared by intervention assignment. Continuous variables were evaluated using t-tests and categorical variables were evaluated with χ^2^ tests. Spearman correlations between individual biomarkers at baseline and change from baseline to the end of the intervention were calculated. Spearman correlations were also calculated for associations between each of the biomarkers and age, BMI, and diabetes duration at baseline and for change between these two times. For each characteristic, we evaluated associations with biomarker levels at baseline and change from baseline to the end of the intervention, adjusted for age and gender using linear regression models. We present these data in two ways, forest plots with each individual measure compared to a ‘standard’ referent category that most resembles existing biomarker studies, and in tables where the least squares means are reported without fixed reference categories.

## Results

There were 779 participants (383 DSE and 396 ILI) with available blood samples and cognitive data for this analysis. Baseline characteristics are shown in Table 1. The mean (standard deviation [SD]) age was 61.4 (6.2); 438 (56.2%) were women; 199 (25.5%) had education <13 years; 130 (16.7%) were African American; and 97 (12.5%) identified as Hispanic. Mean ±SD BMI at baseline was 34.8 ±5.3 kg/m2; 441 (57.2%) had diabetes for at least 5 years; and 668 (86.4%) were taking a diabetes medication. The only difference between randomization groups included in this analysis was in baseline peripheral neuropathy: more participants in the ILI group reported baseline peripheral neuropathy than among those in the DSE group (18.9% vs 12.5%, p=0.01). There were no significant randomization group differences in either baseline or end of intervention levels of blood-based biomarkers (data not shown), thus subsequent analyses of biomarkers were not stratified by intervention arm.

**Table 1.** Baseline Characteristics of Participants by Randomization Group

Characteristic	Total (n= 779)	DSE (n= 383)	ILI (n= 396)	P-value
Age, mean ± SD, years	61.4 ± 6.2	61.3 ± 6.3	61.5 ± 6.0	0.63
Gender, No. (%) Women	438 (56.2%)	219 (57.2%)	219 (55.3%)	0.60
Race, No. (%)				0.65
African American / Black (not Hispanic)	130 (16.7%)	62 (16.2%)	68 (17.2%)	
American Indian / Native American / Alaskan Native	16 (2.1%)	9 (2.3%)	7 (1.8%)	
Asian/Pacific Islander	6 (0.8%)	1 (0.3%)	5 (1.3%)	
Hispanic	97 (12.5%)	48 (12.5%)	49 (12.4%)	
Other/Mixed	14 (1.8%)	8 (2.1%)	6 (1.5%)	
White	516 (66.2%)	255 (66.6%)	261 (65.9%)	
Years of Education, No. (%)				0.28
< 13 years	199 (25.5%)	88 (23.0%)	111 (28.0%)	
13 - 16 years	269 (34.5%)	134 (35.0%)	135 (34.1%)	
> 16 years	288 (37.0%)	148 (38.6%)	140 (35.4%)	
Missing	23 (3.0%)	13 (3.4%)	10 (2.5%)	
APOE ε4 carrier status, No. (%)	151 (22.8%)	67 (21.1%)	84 (24.3%)	0.33
Beck Depression Inventory ≥ 10, No. (%)	129 (16.7%)	61 (16.1%)	68 (17.2%)	0.68
Beck Depression Inventory ≥ 24, No. (%)	5 (0.6%)	3 (0.8%)	2 (0.5%)	0.62
Antidepressant Medication, No. (%)	115 (15.1%)	52 (14.0%)	63 (16.2%)	0.41
Baseline Alcohol Consumption, No. (%)				0.70
None	523 (67.1%)	252 (65.8%)	271 (68.4%)	
< 21 oz/wk	154 (19.8%)	80 (20.9%)	74 (18.7%)	
≥ 21 oz/wk	102 (13.1%)	51 (13.3%)	51 (12.9%)	
Baseline Smoking, No. (%)	22 (2.8%)	8 (2.1%)	14 (3.6%)	0.40
Body Mass Index, mean ± SD, kg/m^2^	34.8 ± 5.3	35.0 ± 5.3	34.6 ± 5.2	0.24
Body Mass Index, No. (%)				0.38
< 30 kg/m^2^	149 (19.1%)	69 (18.0%)	80 (20.2%)	
30-39 kg/m^2^	509 (65.3%)	248 (64.8%)	261 (65.9%)	
40+ kg/m^2^	121 (15.5%)	66 (17.2%)	55 (13.9%)	
Diabetes Duration, No. (%) 5+ yrs	441 (57.2%)	217 (57.3%)	224 (57.1%)	0.98
HbA1c %, mean ± SD	7.2 ± 1.1	7.2 ± 1.2	7.2 ± 1.1	0.82
HbA1c, No. (%)				0.38
< 7%	366 (47.0%)	185 (48.3%)	181 (45.7%)	
7-9%	355 (45.6%)	166 (43.3%)	189 (47.7%)	
≥ 9%	58 (7.4%)	32 (8.4%)	26 (6.6%)	
Baseline Diabetes Treatment, No. (%)				0.98
No Meds	105 (13.6%)	52 (13.7%)	53 (13.5%)	
Oral Meds, No insulin	553 (71.5%)	270 (71.2%)	283 (71.8%)	
Oral Meds + Insulin	115 (14.9%)	57 (15.0%)	58 (14.7%)	
Baseline Peripheral Neuropathy, No. (%)	123 (15.8%)	48 (12.5%)	75 (18.9%)	0.01
eGFR <=60	29 (4.0%)	18 (5.1%)	11 (3.0%)	0.33
eGFR 60-90	292 (40.6%)	139 (39.4%)	153 (41.8%)	
eGFR>=90	398 (55.4%)	196 (55.5%)	202 (55.2%)	
Baseline Hypertension, No. (%)	651 (83.6%)	321 (83.8%)	330 (83.3%)	0.86
Baseline Dyslipidemia, No. (%)	697 (89.5%)	342 (89.3%)	355 (89.6%)	0.87
Baseline CVD History, No. (%)	130 (16.7%)	63 (16.4%)	67 (16.9%)	0.86
Baseline				
Aβ-40, mean ± SD, μg/DL	66.7 ± 19.5	67.0 ± 20.9	66.5 ± 18.0	0.14
Aβ-42, mean ± SD, μg/DL	4.9 ± 1.4	4.9 ± 1.4	4.8 ± 1.4	0.12
Aβ-42/40, mean ± SD, μg/DL	0.1 ± 0.0	0.1 ± 0.0	0.1 ± 0.0	0.60
GFAP, mean ± SD, μg/DL	93.3 ± 40.5	92.5 ± 39.7	94.0 ± 41.4	0.88
NfL, mean ± SD, μg/DL	13.8 ± 6.5	14.3 ± 7.3	13.4 ± 5.5	0.68
pTau-181, mean ± SD, μg/DL	9.2 ± 5.1	9.1 ± 5.0	9.4 ± 5.2	0.20
Years 8-13	N=770	N=377	N=393	
Aβ-40, mean ± SD, μg/DL	89.3 ± 34.8	91.1 ± 36.2	87.4 ± 33.2	0.77
Aβ-42, mean ± SD, μg/DL	6.0 ± 2.3	6.1 ± 2.3	5.9 ± 2.3	0.25
Aβ-42/40, mean ± SD, μg/DL	0.1 ± 0.0	0.1 ± 0.3	0.1 ± 0.0	0.96
GFAP, mean ± SD, μg/DL	153.1 ± 74.7	152.7 ± 71.3	153.5 ± 77.9	0.62
NfL, mean ± SD, μg/DL	25.1 ± 18.7	24.9 ± 18.1	25.4 ± 19.3	0.07
pTau-181, mean ± SD, μg/DL	9.9 ± 5.5	9.7 ± 5.3	10.2 ± 5.6	0.47

Abbreviations Aβ, Amyloid beta; APOE ε4, Apolipoprotein E gene, ε4 carrier status; CVD, cardiovascular disease; DSE, diabetes support and education; GFAP, glial fibrillary acidic protein; eGFR, estimated glomerular filtration rate; HbA1c, hemoglobin A1c; Hx, history; ILI, intensive lifestyle intervention; kg/m^2^, kilogram per square meter; Meds, medications; NfL, neurofilament light chain; oz, ounces; SD, standard deviation, Trt, treatment; μg/DL, microgram per deciliter; wk, week; Yrs, years

Spearman correlations between unadjusted plasma biomarker levels at baseline and change are shown in Tables 2a and 2b. At baseline, all biomarkers significantly correlated with each other, with two exceptions: the Aβ42/40 ratio only correlated with Aβ42 and Aβ40; and ptau181 and GFAP were not correlated. Next, we examined correlations between change in biomarker levels from baseline to the end of the intervention (Table 2b); nearly all biomarkers correlated, except for pTau181 or GFAP with Aβ42/40.

**Table 2a:** Spearman Correlations between Biomarkers at Baseline

Correlation Coefficient Prob > |r| under H_o_: Rho=0 Number of Observations						
	Aβ42	Aβ40	Aβ42/40	pTau181	NfL	GFAP
Aβ42	1.000 - 743					
Aβ40	**0.794** **<.0001** **743**	1.000 - 768				
Aβ42/40	**0.452** **<.0001** **742**	**-0.086** **0.019** **742**	1.000 - 743			
pTau181	**0.094** **0.011** **742**	**0.132** **0.0002** **767**	0.032 0.387 742	1.000 - 769		
NfL	**0.259** **<.0001** **743**	**0.318** **<.0001** **768**	-0.069 0.060 743	**0.165** **<.0001** **769**	1.000 - 770	
GFAP	**0.235** **<.0001** **742**	**0.302** **<.0001** **767**	-0.015 0.676 742	0.057 0.118 768	**0.401** **<.0001** **769**	1.000 - 769

**Table 2b.** Spearman Correlations between Biomarker Change from Baseline to End of the Intervention

Correlation Coefficient Prob > |r| under H_0_: Rho=0 Number of Observations						
	Aβ42	Aβ40	Aβ42/40	pTau181	NfL	GFAP
Aβ42	1.000 - 730					
Aβ40	**0.879** **<.0001** **730**	1.000 - 760				
Aβ42/40	**0.112** **0.003** **728**	**-0.284** **<.0001** **728**	1.000 - 728			
pTau181	**0.142** **0.0001** **728**	**0.147** **<.0001** **758**	0.013 0.738 726	1.000 - 759		
NfL	**0.228** **<.0001** **730**	**0.257** **<.0001** **760**	**-0.103** **0.005** **728**	**0.127** **0.001** **759**	1.000 - 761	
GFAP	**0.252** **<.0001** **729**	**0.269** **<.0001** **759**	-0.051 0.166 727	**0.134** **0.0002** **758**	**0.487** **<.0001** **760**	1.000 - 760

We then examined correlations between baseline biomarker levels and baseline values of age, BMI, and diabetes duration; we also examined correlations between these variables and longitudinal change in biomarker levels (Table 3). At baseline, Aβ42, Aβ40, NfL and GFAP positively correlated with age; Aβ42/40, NfL, and GFAP were inversely correlated with baseline BMI; and diabetes duration positively correlated with NfL. Change in NfL and GFAP positively correlated with age, while change in Aβ42/40 negatively correlated with age.

**Table 3.** Spearman Correlations between Biomarkers, Age, BMI, and Diabetes Duration at Baseline, and Change from Baseline to the End of the Intervention

Correlation Coefficient Prob > |r| under H_0_: Rho=0 Number of Observations							
Baseline and Change from Baseline to End of Intervention							
**Baseline Biomarkers**	**Baseline Age**	**Baseline BMI**	**Diabetes Duration**	**Change**	**Baseline Age**	**Baseline BMI**	**Diabetes Duration**
**Aβ42**	**0.211** **<.0001** **743**	-0.063 0.084 743	0.066 0.074 735	**Aβ42**	-0.065 0.081 730	0.048 0.199 730	0.026 0.494 722
**Aβ40**	**0.293** **<.0001** **768**	-0.058 0.110 768	0.064 0.080 760	**Aβ40**	0.037 0.313 760	0.065 0.075 760	0.040 0.278 752
**Aβ42/40**	-0.051 0.168 743	**-0.100** **0.007** **743**	0.002 0.957 734	**Aβ42/40**	**-0.200** **<.0001** **728**	0.010 0.784 728	-0.055 0.143 720
**pTau181**	0.023 0.522 769	0.021 0.561 769	0.025 0.488 761	**pTau181**	-0.061 0.096 759	-0.035 0.337 759	-0.001 0.977 751
**NfL**	**0.369** **<0.0001** **770**	**-0.166** **<0.001** **770**	**0.233** **<.0001** **762**	**NfL**	**0.150** **<.0001** **761**	-0.018 0.630 761	0.003 0.928 753
**GFAP**	**0.404** **<0.001** **769**	**-0.211** **<0.001** **769**	0.057 0.119 761	**GFAP**	**0.203** **<.0001** **760**	-0.059 0.104 760	0.047 0.196 752

In Figures 2a and 2b, forest plots depict the associations between each biomarker by age groups (45-55, 56-65, 65-76). Both Aβ42 and Aβ40 levels differed significantly among age groups at baseline and change over time with higher biomarker levels in older age groups at baseline and greater increases in biomarker levels over time among the youngest and oldest age groups. Age group was not significantly associated with Aβ42/40 ratio at baseline, but it was associated with change in Aβ42/40 ratio from baseline to years 8-13 (p<0.001). Figure 2b shows no significant differences in pTau181 by age groups at baseline, but significant differences by age group emerged when considering change from baseline (p=0.005). NfL and GFAP demonstrated significant associations with age groups at baseline and change (all p<0.05).

**Figure 2a.**
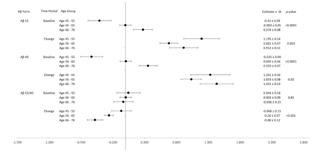
Aβ42, Aβ40 and Aβ42/40 Levels by Age Groups: Baseline and Change over 8-13 years

**Figure 2b.**
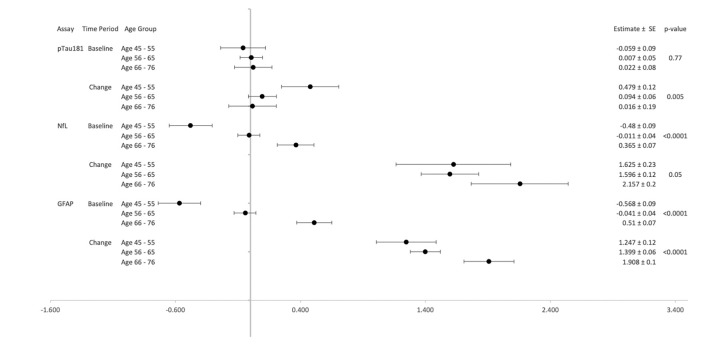
pTau181, NfL, and GFAP Levels by Age Groups: Baseline and Change over 8-13 years

Figure 3a and 3b show forest plots of each biomarker by gender at baseline and change. Women had significantly lower levels of Aβ40 compared to men (p=0.02) at baseline. Women had lower NfL levels compared to men at baseline and change (all p<0.05). However, women had higher levels of GFAP at baseline (p=0.009; Figure 3b).

**Figure 3a.**
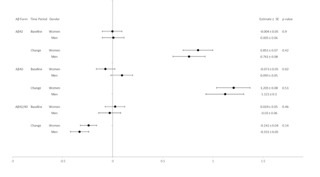
Aβ42, Aβ40 and Aβ42/40 Levels by gender: Baseline and Change over 8-13 years

**Figure 3b.**
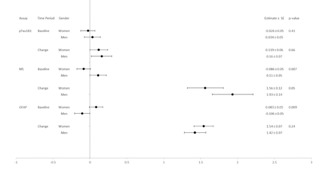
pTau181, NfL, and GFAP Levels by Gender: Baseline and Change over 8-13 years

**Figure 4.**
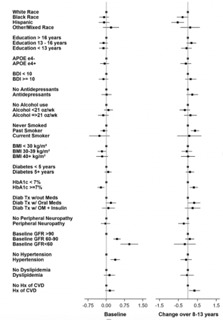
Aβ42 levels at baseline and change over 8-13 years

**Figure 5.**
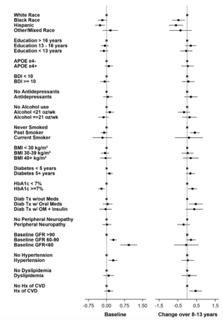
Aβ40 levels at baseline and change over 8-13 years

**Figure 6.**
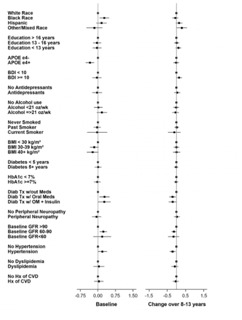
Aβ42/ Aβ40 ratio levels at baseline and change over 8-13 years

**Figure 7.**
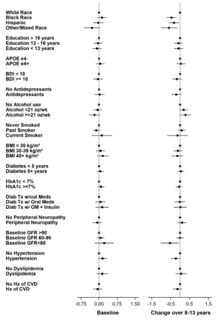
pTau181 levels at baseline and change over 8-13 years

**Figure 8.**
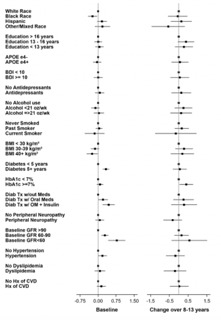
NfL levels at baseline and change over 8-13 years

**Figure 9.**
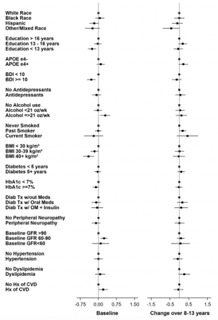
GFAP levels at baseline and change over 8-13 years

Given the effect of age and gender on biomarker levels, subsequent analyses were adjusted for these demographics. Results are presented two ways: 1) visually for baseline and change values (from baseline to end of intervention) as forest plots (Figures 4-9) with comparisons to a common reference category set at zero; and 2) as tables in which least squares means of the z-scores are reported without reference categories and including baseline and change from baseline to the end of the intervention (Tables 4-9).

### Aβ42

Aβ42 levels differed by race and ethnicity in change from baseline (p=0.007); Hispanic and Black participants had lower levels of change than White or other/ mixed race participants (Table 4 and Figure 4). Never smokers had the lowest increase in Aβ42 levels (p=0.004) compared to past or current smokers. Participants with elevated baseline HbA1c levels (≥7%) had lower baseline levels of Aβ42 (p=0.002) and a greater increase over time (p=0.004). Participants with lower eGFR had higher levels of Aβ42 at baseline (p<0.0001). Hypertension at baseline was associated with higher Aβ42 levels (p=0.0002). Those with a history of CVD at baseline had greater increase in Aβ42 over time than those without a baseline history of CVD (p=0.021).

**Table 4.** Aβ42 levels by individual baseline characteristics adjusted for age and gender

	Baseline			Change		
Characteristic	LS Mean	Confidence Interval	p-value	LS Mean	Confidence Interval	p-value
Black Race	-0.074	(-0.252, 0.104)	0.399	**0.638**	**(0.369, 0.907)**	**0.007**
White Race	0.018	(-0.070, 0.105)		**0.919**	**(0.786, 1.051)**	
Hispanic	-0.075	(-0.277, 0.127)		**0.382**	**(0.077, 0.687)**	
Other/Mixed Race	0.209	(-0.111, 0.529)		**1.039**	**(0.543, 1.534)**	
Education < 13 years	-0.048	(-0.189, 0.094)	0.327	0.707	(0.491, 0.923)	0.162
Education 13 - 16 years	-0.047	(-0.168, 0.073)		0.966	(0.781, 1.150)	
Education > 16 years	0.067	(-0.050, 0.184)		0.781	(0.603, 0.959)	
No APOE ε4 alleles	0.027	(-0.061, 0.116)	0.445	0.856	(0.722, 0.989)	0.461
APOE ε4 +	-0.046	(-0.211, 0.120)		0.748	(0.493, 1.002)	
BDI < 10	0.017	(-0.060, 0.093)	0.245	0.797	(0.680, 0.914)	0.325
BDI ≥ 10	-0.100	(-0.280, 0.080)		0.947	(0.673, 1.220)	
No Antidepressants	0.009	(-0.068, 0.087)	0.938	0.767	(0.649, 0.886)	0.136
Antidepressants	0.018	(-0.170, 0.205)		1.001	(0.719, 1.283)	
No alcohol use	-0.000	(-0.088, 0.087)	0.248	0.826	(0.692, 0.959)	0.276
Alcohol <21 oz/wk	0.089	(-0.070, 0.247)		0.660	(0.418, 0.902)	
Alcohol ≥21 oz/wk	-0.124	(-0.322, 0.074)		0.961	(0.660, 1.261)	
Never Smoked	0.057	(-0.043, 0.156)	0.221	**0.637**	**(0.485, 0.788)**	**0.004**
Past Smoker	-0.043	(-0.149, 0.063)		**1.013**	**(0.852, 1.174)**	
Current Smoker	-0.227	(-0.640, 0.185)		**0.709**	**(0.088, 1.329)**	
BMI < 30 kg/m^2^	0.143	(-0.017, 0.304)	0.152	0.846	(0.600, 1.091)	0.880
BMI 30-39 kg/m^2^	-0.035	(-0.122, 0.052)		0.791	(0.657, 0.924)	
BMI 40+ kg/m^2^	-0.027	(-0.208, 0.153)		0.853	(0.580, 1.127)	
Diabetes < 5 years	-0.036	(-0.145, 0.073)	0.317	0.738	(0.572, 0.905)	0.324
Diabetes 5+ years	0.038	(-0.056, 0.131)		0.849	(0.706, 0.992)	
HbA1c < 7%	**0.117**	**(0.015, 0.219)**	**0.002**	**0.647**	**(0.492, 0.803)**	**0.004**
HbA1c ≥7%	**-0.104**	**(-0.201, -0.008)**		**0.960**	**(0.812, 1.108)**	
Diabetes Trt w/out Meds	-0.142	(-0.336, 0.053)	0.163	0.528	(0.229, 0.827)	0.137
Diabetes Trt w/ Oral Meds, No Insulin	0.048	(-0.036, 0.131)		0.856	(0.730, 0.983)	
Diabetes Trt w/ OM + Insulin	-0.057	(-0.239, 0.125)		0.779	(0.501, 1.057)	
No Peripheral Neuropathy	0.006	(-0.071, 0.083)	0.740	0.846	(0.728, 0.963)	0.150
Peripheral Neuropathy	-0.027	(-0.204, 0.150)		0.630	(0.360, 0.899)	
Baseline GFR ≤ 60	**0.742**	**(0.393, 1.092)**	**<.0001**	0.595	(0.046, 1.145)	0.542
Baseline GFR 60-90	**0.238**	**(0.126, 0.350)**		0.859	(0.682, 1.035)	
Baseline GFR ≥ 90	**-0.204**	**(-0.301, -0.106)**		0.760	(0.606, 0.914)	
No hypertension	**-0.308**	**(-0.485, -0.131)**	**0.0002**	0.888	(0.614, 1.163)	0.549
Hypertension	**0.059**	**(-0.017, 0.135)**		0.797	(0.680, 0.914)	
No Dyslipidemia	-0.114	(-0.333, 0.105)	0.277	0.848	(0.512, 1.184)	0.820
Dyslipidemia	0.014	(-0.061, 0.088)		0.807	(0.693, 0.921)	
No Hx of CVD	-0.023	(-0.101, 0.054)	0.148	**0.753**	**(0.634, 0.871)**	**0.021**
Hx of CVD	0.120	(-0.057, 0.297)		**1.101**	**(0.833, 1.369)**	

Each categorical group of characteristics was evaluated individually, controlling for age and gender. Significant associations are bolded; p-values are presented for each group. Abbreviations: Aβ, Amyloid beta; APOE ε4, Apolipoprotein E gene, ε4 carrier status; BDI, Beck Depression Inventory; BMI, body mass index; CVD, cardiovascular disease; GFR, glomerular filtration rate; HbA1c, hemoglobin A1c; Hx, history; kg/m^2^, kilogram per meters squared; LDL, low density lipoprotein; LS, least-squares; OM, oral medication; oz, ounces; Trt, treatment; wk, week; w/, with; w/out, without; Yrs, years

### Aβ40

White participants and those reporting other/mixed race and ethnicity had higher Aβ40 levels than Black or Hispanic participants at baseline (p=0.018); and greater increases from baseline (p=0.003) (Table 5 and Figure 5). Participants who never smoked had had less of an increase in Aβ40 over time (p=0.008) than past or current smokers. Participants with HbA1c levels ≥7% had lower Aβ40 levels at baseline (p=0.002) and a greater increase over time (p=0.025). Participants with lower baseline eGFR had higher levels of Aβ40 (p<0.0001) at baseline compared to those with higher eGFR. Participants with hypertension at baseline had higher levels of Aβ40 at baseline than those without hypertension (p=0.004); participants with a baseline history of CVD had a greater increase in Aβ40 levels than those without a baseline history of CVD (p=0.009).

**Table 5.** Aβ40 levels by individual baseline characteristics adjusted for age and gender

	Baseline			Change		
Characteristic	LS Mean	Confidence Interval	p-value	LS Mean	Confidence Interval	p-value
Black Race	**-0.119**	**(-0.290, 0.052)**	**0.018**	**0.869**	**(0.560, 1.178)**	**0.003**
White Race	**0.056**	**(-0.028, 0.140)**		**1.338**	**(1.186, 1.489)**	
Hispanic	**-0.218**	**(-0.408, -0.027)**		**0.739**	**(0.395, 1.082)**	
Other/Mixed Race	**0.213**	**(-0.098, 0.525)**		**0.980**	**(0.402, 1.558)**	
Education < 13 years	-0.065	(-0.200, 0.070)	0.447	1.079	(0.833, 1.325)	0.275
Education 13 - 16 years	-0.022	(-0.137, 0.094)		1.319	(1.108, 1.530)	
Education > 16 years	0.047	(-0.066, 0.159)		1.129	(0.924, 1.334)	
No APOE ε4 alleles	0.001	(-0.081, 0.084)	0.285	1.172	(1.018, 1.325)	0.893
APOE ε4 +	0.095	(-0.056, 0.247)		1.194	(0.909, 1.479)	
BDI < 10	0.015	(-0.059, 0.089)	0.430	1.168	(1.033, 1.303)	0.734
BDI ≥ 10	-0.061	(-0.233, 0.112)		1.227	(0.914, 1.541)	
No Antidepressants	0.001	(-0.074, 0.077)	0.621	1.133	(0.997, 1.269)	0.282
Antidepressants	0.051	(-0.130, 0.232)		1.328	(1.002, 1.653)	
No alcohol use	-0.010	(-0.095, 0.074)	0.096	1.185	(1.032, 1.338)	0.386
Alcohol <21 oz/wk	0.126	(-0.027, 0.279)		1.014	(0.735, 1.293)	
Alcohol ≥21 oz/wk	-0.135	(-0.328, 0.058)		1.309	(0.958, 1.660)	
Never Smoked	0.053	(-0.044, 0.149)	0.316	**0.979**	**(0.805, 1.153)**	**0.008**
Past Smoker	-0.048	(-0.150, 0.054)		**1.386**	**(1.201, 1.570)**	
Current Smoker	-0.121	(-0.524, 0.282)		**1.071**	**(0.345, 1.796)**	
BMI < 30 kg/m^2^	0.046	(-0.110, 0.202)	0.779	1.126	(0.844, 1.407)	0.585
BMI 30-39 kg/m^2^	-0.016	(-0.100, 0.068)		1.144	(0.991, 1.297)	
BMI 40+ kg/m^2^	0.011	(-0.162, 0.184)		1.318	(1.006, 1.630)	
Diabetes < 5 years	-0.068	(-0.172, 0.036)	0.087	1.072	(0.882, 1.262)	0.194
Diabetes 5+ years	0.053	(-0.037, 0.143)		1.239	(1.074, 1.403)	
HbA1c < 7%	**0.113**	**(0.015, 0.212)**	**0.002**	**1.018**	**(0.839, 1.198)**	**0.025**
HbA1c ≥7%	**-0.101**	**(-0.194, -0.008)**		**1.301**	**(1.132, 1.470)**	
Diabetes Trt w/out Meds	-0.019	(-0.205, 0.168)	0.688	0.776	(0.438, 1.114)	0.053
Diabetes Trt w/ Oral Meds, No Insulin	0.023	(-0.057, 0.104)		1.231	(1.085, 1.376)	
Diabetes Trt w/ OM + Insulin	-0.058	(-0.234, 0.118)		1.151	(0.831, 1.471)	
No Peripheral Neuropathy	-0.002	(-0.076, 0.072)	0.910	1.198	(1.064, 1.333)	0.262
Peripheral Neuropathy	0.009	(-0.161, 0.179)		1.005	(0.695, 1.315)	
Baseline GFR ≤ 60	**0.786**	**(0.448, 1.124)**	**<.0001**	1.006	(0.372, 1.639)	0.296
Baseline GFR 60-90	**0.144**	**(0.037, 0.251)**		1.263	(1.062, 1.464)	
Baseline GFR ≥ 90	**-0.141**	**(-0.234, -0.049)**		1.059	(0.886, 1.232)	
No hypertension	**-0.232**	**(-0.402, -0.062)**	**0.004**	1.147	(0.835, 1.459)	0.886
Hypertension	**0.044**	**(-0.030, 0.118)**		1.172	(1.037, 1.307)	
No Dyslipidemia	-0.075	(-0.287, 0.136)	0.461	1.074	(0.687, 1.461)	0.615
Dyslipidemia	0.009	(-0.063, 0.080)		1.179	(1.049, 1.309)	
No Hx of CVD	-0.016	(-0.091, 0.059)	0.318	**1.092**	**(0.956, 1.227)**	**0.009**
Hx of CVD	0.079	(-0.090, 0.249)		**1.541**	**(1.235, 1.847)**	

Each categorical group of characteristics was evaluated individually, controlling for age and gender. Significant associations are bolded; p-values are presented for each group. Abbreviations: Aβ, Amyloid beta; APOE ε4, Apolipoprotein E gene, ε4 carrier status; BDI, Beck Depression Inventory; BMI, body mass index; CVD, cardiovascular disease; GFR, glomerular filtration rate; HbA1c, hemoglobin A1c; Hx, history; kg/m^2^, kilogram per meters squared; LDL, low density lipoprotein; LS, least-squares; OM, oral medication; oz, ounces; Trt, treatment; wk, week; w/, with; w/out, without; Yrs, years

### Aβ42/40

At baseline, Black participants had a higher Aβ42/40 ratio compared to White participants and those reporting other/mixed race (p=0.029) (Table 6 and Figure 6). APOE ε4 carriers had a lower Aβ42/40 ratio at baseline (p=0.003). Participants who had diabetes treated with medications had a higher Aβ42/40 ratio compared to those who were not treated with oral medications or insulin (p=0.038). Higher eGFR was associated with a lower Aβ42/40 ratio at baseline (p=0.018). Participants who self-reported hypertension at baseline had greater decrease in Aβ42/40 ratio compared to those who did not (p=0.012).

**Table 6.** Aβ42/Aβ40 levels by individual baseline characteristics adjusted for age and gender

	Baseline			Change		
Characteristic	LS Mean	Confidence Interval	p-value	LS Mean	Confidence Interval	p-value
Black Race	**0.216**	**(0.034, 0.397)**	**0.029**	-0.366	(-0.514, - 0.218)	0.051
White Race	**-0.057**	**(-0.146, 0.033)**		-0.303	(-0.376, -0.231)	
Hispanic	**0.097**	**(-0.108, 0.303)**		-0.155	(-0.321, 0.012)	
Other/Mixed Race	**-0.207**	**(-0.538, 0.124)**		-0.004	(-0.279, 0.271)	
Education < 13 years	-0.016	(-0.162, 0.130)	0.893	-0.205	(-0.322, -0.087)	0.330
Education 13 - 16 years	-0.014	(-0.138, 0.111)		-0.302	(-0.403, -0.201)	
Education > 16 years	0.023	(-0.098, 0.144)		-0.315	(-0.412, -0.218)	
No APOE ε4 alleles	**0.069**	**(-0.018, 0.156)**	**0.003**	-0.279	(-0.352, -0.206)	0.854
APOE ε4 +	**-0.275**	**(-0.438, -0.112)**		-0.294	(-0.432, -0.155)	
BDI < 10	0.004	(-0.075, 0.082)	0.671	-0.302	(-0.366, -0.238)	0.079
BDI ≥ 10	-0.040	(-0.223, 0.144)		-0.156	(-0.306, -0.007)	
No Antidepressants	0.004	(-0.075, 0.084)	0.745	-0.291	(-0.356, -0.227)	0.571
Antidepressants	-0.030	(-0.222, 0.162)		-0.243	(-0.397, -0.089)	
No alcohol use	-0.026	(-0.115, 0.064)	0.331	-0.272	(-0.345, -0.199)	0.887
Alcohol <21 oz/wk	-0.010	(-0.172, 0.152)		-0.288	(-0.420, -0.156)	
Alcohol ≥21 oz/wk	0.144	(-0.059, 0.346)		-0.317	(-0.481, -0.153)	
Never Smoked	0.016	(-0.086, 0.118)	0.920	-0.285	(-0.369, -0.202)	0.757
Past Smoker	-0.015	(-0.124, 0.094)		-0.268	(-0.356, -0.179)	
Current Smoker	-0.015	(-0.438, 0.408)		-0.399	(-0.739, -0.059)	
BMI < 30 kg/m^2^	0.139	(-0.026, 0.304)	0.152	-0.284	(-0.419, -0.150)	0.994
BMI 30-39 kg/m^2^	-0.019	(-0.108, 0.070)		-0.282	(-0.355, -0.209)	
BMI 40+ kg/m^2^	-0.089	(-0.274, 0.095)		-0.274	(-0.423, -0.125)	
Diabetes < 5 years	0.002	(-0.110, 0.113)	0.931	-0.282	(-0.373, -0.191)	0.800
Diabetes 5+ years	0.008	(-0.088, 0.104)		-0.298	(-0.375, -0.220)	
HbA1c < 7%	0.015	(-0.090, 0.120)	0.715	-0.273	(-0.358, -0.188)	0.788
HbA1c ≥7%	-0.012	(-0.112, 0.087)		-0.289	(-0.370, -0.208)	
Diabetes Trt w/out Meds	**-0.241**	**(-0.439, -0.042)**	**0.038**	-0.100	(-0.262, 0.063)	0.063
Diabetes Trt w/ Oral Meds, No Insulin	**0.040**	**(-0.045, 0.125)**		-0.310	(-0.379, -0.240)	
Diabetes Trt w/ OM + Insulin	**0.030**	**(-0.155, 0.216)**		-0.307	(-0.458, -0.156)	
No Peripheral Neuropathy	0.010	(-0.069, 0.088)	0.567	-0.286	(-0.350, -0.222)	0.705
Peripheral Neuropathy	-0.048	(-0.229, 0.133)		-0.255	(-0.402, -0.108)	
Baseline GFR ≤ 60	**0.090**	**(-0.281, 0.461)**	**0.018**	-0.381	(-0.677, -0.084)	0.094
Baseline GFR 60-90	**0.137**	**(0.018, 0.256)**		-0.361	(-0.456, -0.265)	
Baseline GFR ≥ 90	**-0.092**	**(-0.195, 0.012)**		-0.225	(-0.308, -0.142)	
No hypertension	-0.159	(-0.341, 0.023)	0.062	**-0.105**	**(-0.254, 0.044)**	**0.012**
Hypertension	0.031	(-0.048, 0.109)		**-0.314**	**(-0.378, -0.250)**	
No Dyslipidemia	-0.035	(-0.259, 0.189)	0.745	-0.202	(-0.385, -0.019)	0.370x
Dyslipidemia	0.004	(-0.072, 0.081)		-0.291	(-0.352, -0.229)	
No Hx of CVD	-0.006	(-0.085, 0.074)	0.708	-0.275	(-0.340, -0.211)	0.652
Hx of CVD	0.032	(-0.149, 0.213)		-0.312	(-0.460, -0.165)	

Each categorical group of characteristics was evaluated individually, controlling for age and gender. Significant associations are bolded; p-values are presented for each group. Abbreviations: Aβ, Amyloid beta; APOE ε4, Apolipoprotein E gene, ε4 carrier status; BDI, Beck Depression Inventory; BMI, body mass index; CVD, cardiovascular disease; GFR, glomerular filtration rate; HbA1c, hemoglobin A1c; Hx, history; kg/m^2^, kilogram per meters squared; LDL, low density lipoprotein; LS, least-squares; OM, oral medication; oz, ounces; Trt, treatment; wk, week; w/, with; w/out, without; Yrs, years

### pTau181

At baseline, there were no significant associations between any characteristic and ptau181 levels, adjusting for age and gender (Table 7 and Figure 7). In the time from baseline to the end of the intervention, White participants had the greatest increase in pTau181 levels over time compared to the other groups (p=0.0002).

**Table 7:** Ptau181 levels by individual baseline characteristics adjusted for age and gender

	Baseline			Change		
Characteristic	LS Mean	Confidence Interval	p-value	LS Mean	Confidence Interval	p-value
Black Race	0.117	(-0.063, 0.296)	0.181	**-0.180**	**(-0.405, 0.046)**	**0.0002**
White Race	-0.002	(-0.090, 0.086)		**0.279**	**(0.169, 0.390)**	
Hispanic	-0.031	(-0.231, 0.169)		**-0.045**	**(-0.296, 0.205)**	
Other/Mixed Race	-0.296	(-0.623, 0.031)		**-0.308**	**(-0.729, 0.114)**	
Education < 13 years	-0.051	(-0.193, 0.091)	0.678	0.116	(-0.063, 0.296)	0.654
Education 13 - 16 years	-0.008	(-0.130, 0.113)		0.116	(-0.039, 0.270)	
Education > 16 years	0.032	(-0.086, 0.151)		0.206	(0.056, 0.355)	
No APOE ε4 alleles	-0.017	(-0.105, 0.072)	0.610	0.176	(0.064, 0.287)	0.768
APOE ε4 +	0.031	(-0.132, 0.194)		0.211	(0.003, 0.419)	
BDI < 10	0.006	(-0.071, 0.084)	0.647	0.168	(0.070, 0.267)	0.174
BDI ≥ 10	-0.040	(-0.221, 0.141)		-0.005	(-0.234, 0.224)	
No Antidepressants	-0.001	(-0.078, 0.077)	0.834	0.183	(0.084, 0.282)	0.114
Antidepressants	0.021	(-0.166, 0.208)		-0.024	(-0.259, 0.212)	
No alcohol use	0.037	(-0.051, 0.125)	0.247	0.062	(-0.050, 0.174)	0.076
Alcohol <21 oz/wk	-0.022	(-0.182, 0.137)		0.248	(0.045, 0.452)	
Alcohol ≥21 oz/wk	-0.153	(-0.356, 0.050)		0.349	(0.092, 0.607)	
Never Smoked	0.051	(-0.050, 0.151)	0.270	0.100	(-0.028, 0.229)	0.578
Past Smoker	-0.058	(-0.164, 0.048)		0.181	(0.046, 0.317)	
Current Smoker	0.162	(-0.259, 0.583)		-0.034	(-0.568, 0.499)	
BMI < 30 kg/m^2^	-0.065	(-0.229, 0.099)	0.448	0.239	(0.032, 0.446)	0.549
BMI 30-39 kg/m^2^	-0.003	(-0.091, 0.084)		0.117	(0.006, 0.229)	
BMI 40+ kg/m^2^	0.092	(-0.088, 0.271)		0.092	(-0.137, 0.321)	
Diabetes < 5 years	-0.028	(-0.137, 0.081)	0.481	0.114	(-0.025, 0.253)	0.669
Diabetes 5+ years	0.024	(-0.070, 0.118)		0.154	(0.034, 0.275)	
HbA1c < 7%	-0.044	(-0.148, 0.059)	0.247	0.166	(0.034, 0.297)	0.555
HbA1c ≥7%	0.040	(-0.058, 0.137)		0.111	(-0.013, 0.235)	
Diabetes Trt w/out Meds	-0.059	(-0.253, 0.135)	0.629	0.152	(-0.097, 0.401)	0.974
Diabetes Trt w/ Oral Meds, No Insulin	0.000	(-0.084, 0.084)		0.136	(0.029, 0.243)	
Diabetes Trt w/ OM + Insulin	0.072	(-0.113, 0.256)		0.164	(-0.072, 0.401)	
No Peripheral Neuropathy	0.011	(-0.066, 0.088)	0.477	0.120	(0.022, 0.218)	0.409
Peripheral Neuropathy	-0.059	(-0.237, 0.119)		0.224	(-0.003, 0.451)	
Baseline GFR ≤ 60	0.213	(-0.159, 0.585)	0.457	-0.428	(-0.894, 0.037)	0.086
Baseline GFR 60-90	0.061	(-0.057, 0.178)		0.105	(-0.043, 0.252)	
Baseline GFR ≥ 90	-0.004	(-0.105, 0.098)		0.113	(-0.014, 0.240)	
No hypertension	-0.123	(-0.301, 0.056)	0.143	0.327	(0.099, 0.555)	0.074
Hypertension	0.023	(-0.054, 0.101)		0.101	(0.002, 0.199)	
No Dyslipidemia	-0.118	(-0.339, 0.103)	0.268	0.091	(-0.194, 0.376)	0.740
Dyslipidemia	0.014	(-0.061, 0.088)		0.142	(0.047, 0.237)	
No Hx of CVD	0.009	(-0.069, 0.088)	0.579	0.160	(0.061, 0.260)	0.276
Hx of CVD	-0.046	(-0.223, 0.131)		0.022	(-0.202, 0.247)	

Each categorical group of characteristics was evaluated individually, controlling for age and gender. Significant associations are bolded; p-values are presented for each group. Abbreviations: Aβ, Amyloid beta; APOE ε4, Apolipoprotein E gene, ε4 carrier status; BDI, Beck Depression Inventory; BMI, body mass index; CVD, cardiovascular disease; GFR, glomerular filtration rate; HbA1c, hemoglobin A1c; Hx, history; kg/m^2^, kilogram per meters squared; LDL, low density lipoprotein; LS, least-squares; OM, oral medication; oz, ounces; Trt, treatment; wk, week; w/, with; w/out, without; Yrs, years

### NfL

Baseline NfL levels (Table 8 and Figure 8), adjusted for age and gender, are associated with race and ethnicity, with Black participants having the lowest NfL levels at baseline (p=0.010). Participants who had diabetes <5 years at baseline had lower NfL levels than those who had diabetes ≥5 years (p<0.0001). Participants with lower HbA1c levels at baseline had less increase in NfL levels by the end of the intervention (p=0.032). Participants who treated their diabetes without medication had the lowest NfL levels at baseline (p=0.003). Lower eGFR levels were associated with higher NfL levels at baseline (p<0.0001).

**Table 8.** NfL levels by individual baseline characteristics adjusted for age and gender

	Baseline			Change		
Characteristic	LS Mean	Confidence Interval	p-value	LS Mean	Confidence Interval	p-value
Black Race	**-0.224**	**(-0.394, -0.054)**	**0.010**	1.684	(1.225, 2.143)	0.581
White Race	**0.009**	**(-0.074, 0.093)**		1.743	(1.517, 1.968)	
Hispanic	**0.171**	**(-0.019, 0.360)**		1.879	(1.368, 2.389)	
Other/Mixed Race	**0.191**	**(-0.119, 0.502)**		1.180	(0.321, 2.040)	
Education < 13 years	0.038	(-0.095, 0.170)	0.809	1.817	(1.451, 2.182)	0.214
Education 13 - 16 years	-0.011	(-0.125, 0.102)		1.926	(1.612, 2.240)	
Education > 16 years	-0.016	(-0.126, 0.095)		1.542	(1.237, 1.846)	
No APOE ε4 alleles	0.003	(-0.080, 0.086)	0.646	1.707	(1.492, 1.921)	0.782
APOE ε4 +	-0.038	(-0.191, 0.115)		1.770	(1.372, 2.169)	
BDI < 10	0.000	(-0.074, 0.074)	0.966	1.722	(1.523, 1.921)	0.842
BDI ≥ 10	0.004	(-0.169, 0.177)		1.774	(1.310, 2.237)	
No Antidepressants	-0.005	(-0.080, 0.070)	0.503	1.720	(1.518, 1.922)	0.629
Antidepressants	0.062	(-0.118, 0.241)		1.850	(1.367, 2.332)	
No alcohol use	0.018	(-0.066, 0.102)	0.378	1.660	(1.434, 1.885)	0.575
Alcohol <21 oz/wk	-0.094	(-0.246, 0.058)		1.909	(1.498, 2.321)	
Alcohol ≥21 oz/wk	0.051	(-0.142, 0.243)		1.784	(1.266, 2.301)	
Never Smoked	-0.011	(-0.106, 0.085)	0.623	1.660	(1.402, 1.918)	0.649
Past Smoker	0.030	(-0.071, 0.131)		1.821	(1.548, 2.094)	
Current Smoker	-0.156	(-0.558, 0.245)		1.501	(0.425, 2.576)	
BMI < 30 kg/m^2^	0.124	(-0.031, 0.279)	0.103	1.511	(1.095, 1.926)	0.496
BMI 30-39 kg/m^2^	-0.006	(-0.089, 0.078)		1.794	(1.569, 2.019)	
BMI 40+ kg/m^2^	-0.126	(-0.297, 0.044)		1.702	(1.243, 2.162)	
Diabetes < 5 years	**-0.188**	**(-0.291, -0.086)**	**<.0001**	1.699	(1.418, 1.979)	0.783
Diabetes 5+ years	**0.139**	**(0.051, 0.228)**		1.751	(1.508, 1.994)	
HbA1c < 7%	-0.042	(-0.140, 0.057)	0.253	**1.514**	**(1.250, 1.779)**	**0.032**
HbA1c ≥7%	0.037	(-0.056, 0.130)		**1.913**	**(1.663, 2.162)**	
Diabetes Trt w/out Meds	**-0.214**	**(-0.398, -0.031)**	**0.003**	1.505	(1.003, 2.008)	0.635
Diabetes Trt w/ Oral Meds, No Insulin	**-0.008**	**(-0.087, 0.072)**		1.761	(1.545, 1.976)	
Diabetes Trt w/ OM + Insulin	**0.225**	**(0.051, 0.399)**		1.790	(1.314, 2.265)	
No Peripheral Neuropathy	-0.011	(-0.084, 0.063)	0.475	1.777	(1.579, 1.974)	0.200
Peripheral Neuropathy	0.057	(-0.113, 0.226)		1.451	(0.995, 1.908)	
Baseline GFR ≤ 60	**0.629**	**(0.284, 0.973)**	**<.0001**	2.181	(1.265, 3.096)	0.441
Baseline GFR 60-90	**0.133**	**(0.024, 0.242)**		1.761	(1.471, 2.052)	
Baseline GFR ≥ 90	**-0.149**	**(-0.243, -0.055)**		1.615	(1.366, 1.865)	
No hypertension	-0.153	(-0.322, 0.017)	0.054	1.877	(1.419, 2.335)	0.479
Hypertension	0.029	(-0.044, 0.103)		1.696	(1.498, 1.895)	
No Dyslipidemia	-0.069	(-0.278, 0.141)	0.497	1.907	(1.337, 2.478)	0.509
Dyslipidemia	0.008	(-0.063, 0.079)		1.705	(1.513, 1.896)	
No Hx of CVD	-0.023	(-0.097, 0.052)	0.159	1.721	(1.521, 1.922)	0.923
Hx of CVD	0.111	(-0.058, 0.280)		1.746	(1.292, 2.199)	

Each categorical group of characteristics was evaluated individually, controlling for age and gender. Significant associations are bolded; p-values are presented for each group. Abbreviations: Aβ, Amyloid beta; APOE ε4, Apolipoprotein E gene, ε4 carrier status; BDI, Beck Depression Inventory; BMI, body mass index; CVD, cardiovascular disease; GFR, glomerular filtration rate; HbA1c, hemoglobin A1c; Hx, history; kg/m^2^, kilogram per meters squared; LDL, low density lipoprotein; LS, least-squares; OM, oral medication; oz, ounces; Trt, treatment; wk, week; w/, with; w/out, without; Yrs, years

### GFAP

Lower GFAP levels (Table 9, Figure 9) were associated with lower education levels at baseline (p=0.006). BDI scores ≥10 were associated with lower GFAP levels at baseline (p=0.001). Alcohol use at baseline ≥21 oz/week was associated with higher GFAP levels at baseline (p=0.019). Higher baseline BMI was associated with lower GFAP levels at baseline (p=0.002). Participants with baseline eGFR of 60-90 had the highest GFAP levels at baseline (p=0.006). Finally, having a history of CVD at baseline was associated with higher GFAP levels at baseline (p=0.032).

**Table 9.** GFAP levels by individual baseline characteristics adjusted for age and gender

	Baseline			Change		
Characteristic	LS Mean	Confidence Interval	p-value	LS Mean	Confidence Interval	p-value
Black Race	0.081	(-0.085, 0.247)	0.110	1.520	(1.282, 1.758)	0.292
White Race	0.024	(-0.057, 0.106)		1.521	(1.404, 1.638)	
Hispanic	-0.153	(-0.338, 0.032)		1.415	(1.150, 1.679)	
Other/Mixed Race	-0.224	(-0.527, 0.079)		1.092	(0.647, 1.537)	
Education < 13 years	**-0.188**	**(-0.319, -0.056)**	**0.006**	1.391	(1.202, 1.580)	0.124
Education 13 - 16 years	**0.050**	**(-0.063, 0.163)**		1.631	(1.468, 1.793)	
Education > 16 years	**0.076**	**(-0.034, 0.185)**		1.451	(1.294, 1.608)	
No APOE ε4 alleles	-0.037	(-0.117, 0.043)	0.256	1.439	(1.322, 1.557)	0.098
APOE ε4 +	0.060	(-0.087, 0.206)		1.649	(1.431, 1.867)	
BDI < 10	**0.044**	**(-0.027, 0.115)**	**0.001**	1.510	(1.407, 1.613)	0.304
BDI ≥ 10	**-0.253**	**(-0.418, -0.087)**		1.373	(1.133, 1.613)	
No Antidepressants	0.019	(-0.053, 0.092)	0.423	1.490	(1.386, 1.595)	0.702
Antidepressants	-0.058	(-0.233, 0.116)		1.544	(1.294, 1.793)	
No alcohol use	**-0.033**	**(-0.115, 0.048)**	**0.019**	1.466	(1.349, 1.583)	0.768
Alcohol <21 oz/wk	**-0.054**	**(-0.202, 0.093)**		1.506	(1.293, 1.720)	
Alcohol ≥21 oz/wk	**0.248**	**(0.062, 0.435)**		1.574	(1.305, 1.842)	
Never Smoked	0.011	(-0.082, 0.104)	0.634	1.396	(1.263, 1.530)	0.100
Past Smoker	0.002	(-0.097, 0.101)		1.573	(1.432, 1.715)	
Current Smoker	-0.185	(-0.576, 0.207)		1.828	(1.272, 2.384)	
BMI < 30 kg/m^2^	**0.170**	**(0.020, 0.320)**	**0.002**	1.489	(1.273, 1.704)	0.965
BMI 30-39 kg/m^2^	**0.008**	**(-0.072, 0.089)**		1.495	(1.378, 1.612)	
BMI 40+ kg/m^2^	**-0.242**	**(-0.407, -0.077)**		1.459	(1.221, 1.698)	
Diabetes < 5 years	0.022	(-0.079, 0.123)	0.673	1.435	(1.290, 1.581)	0.306
Diabetes 5+ years	-0.007	(-0.095, 0.081)		1.536	(1.410, 1.662)	
HbA1c < 7%	0.055	(-0.041, 0.151)	0.119	1.498	(1.360, 1.636)	0.848
HbA1c ≥7%	-0.050	(-0.140, 0.041)		1.480	(1.350, 1.609)	
Diabetes Trt w/out Meds	0.072	(-0.107, 0.252)	0.640	1.358	(1.098, 1.618)	0.522
Diabetes Trt w/ Oral Meds, No Insulin	-0.019	(-0.097, 0.059)		1.518	(1.407, 1.630)	
Diabetes Trt w/ OM + Insulin	0.017	(-0.154, 0.187)		1.457	(1.211, 1.703)	
No Peripheral Neuropathy	0.016	(-0.056, 0.087)	0.279	1.487	(1.385, 1.590)	0.969
Peripheral Neuropathy	-0.084	(-0.249, 0.081)		1.493	(1.255, 1.730)	
Baseline GFR ≤ 60	**-0.014**	**(-0.355, 0.327)**	**0.006**	1.487	(1.010, 1.965)	0.722
Baseline GFR 60-90	**0.128**	**(0.020, 0.237)**		1.510	(1.359, 1.662)	
Baseline GFR ≥ 90	**-0.105**	**(-0.199, -0.012)**		1.428	(1.297, 1.558)	
No hypertension	-0.002	(-0.167, 0.164)	0.986	1.556	(1.318, 1.793)	0.543
Hypertension	0.000	(-0.072, 0.072)		1.475	(1.372, 1.578)	
No Dyslipidemia	-0.064	(-0.268, 0.139)	0.514	1.254	(0.958, 1.549)	0.101
Dyslipidemia	0.007	(-0.062, 0.077)		1.515	(1.415, 1.614)	
No Hx of CVD	**-0.034**	**(-0.106, 0.039)**	**0.032**	1.496	(1.392, 1.600)	0.719
Hx of CVD	**0.165**	**(0.000, 0.329)**		1.449	(1.213, 1.685)	

Each categorical group of characteristics was evaluated individually, controlling for age and gender. Significant associations are bolded; p-values are presented for each group. Abbreviations: Aβ, Amyloid beta; APOE ε4, Apolipoprotein E gene, ε4 carrier status; BDI, Beck Depression Inventory; BMI, body mass index; CVD, cardiovascular disease; GFR, glomerular filtration rate; HbA1c, hemoglobin A1c; Hx, history; kg/m^2^, kilogram per meters squared; LDL, low density lipoprotein; LS, least-squares; OM, oral medication; oz, ounces; Trt, treatment; wk, week; w/, with; w/out, without; Yrs, years

### Intervention

As noted above, there were no significant intervention group differences at baseline in biomarker levels. Change in biomarker levels from baseline to the end of the intervention also did not differ by intervention arm (Table 1). Interactions between intervention arm and age, gender, BMI, history of CVD, and APOE ε4 carrier status were tested. There was a significant interaction between intervention group and age such that those over age 65 at baseline and randomized to DSE had greater increases in NfL than those under age 65. Those over age 65 at baseline and randomized to DSE also had a greater increase in NfL than those in the ILI group who were aged 65 or older. No other interactions by randomization group emerged.

## Discussion

Blood-based AD biomarkers have complex associations with each other, with age and gender, and with a number of common conditions that are themselves risk factors for dementia. Some of these conditions may influence blood biomarker levels because they are risk factors for AD whereas others may influence the biomarker levels because they physiologically impact peripheral levels (4, 8). Most studies of blood-based AD biomarkers conducted to date, have focused on relatively healthy and homogenous populations which do not reflect the reality of older populations with multiple chronic conditions and cognitive impairment, who will be most likely to have blood biomarker testing. There is an urgent need to understand the impact of factors that may affect the interpretation of the blood markers in the general population, especially now that some of these biomarkers are clinically available for use in forming diagnoses at the population level. In this study, we examined associations between AD-related plasma biomarkers (Aβ42, Aβ40, Aβ42/Aβ40, pTau181, NfL, and GFAP) and common conditions in older adults with T2D and overweight or obesity. The cohort comprised clinical trial participants, so we also evaluated the legacy effect of the intensive lifestyle intervention on biomarker levels. Of the 18 factors tested, 15 had significant associations with at least one of the AD blood-based biomarkers. We discuss and place into context our results for each factor studied below. We have chosen to focus on comparisons to studies with similar methods, age ranges, and those that mostly included participants who were cognitively normal at their baseline assessment. See Supplemental Table 2 for basic information on the comparison studies including biomarker methods, biomarkers targeted, sample size, age, and cognitive status.

### Age

At baseline, Aβ42, Aβ40, NfL, and GFAP were positively associated with age. At the end of the intervention, Aβ42/Aβ40 was inversely associated with age, while NfL and GFAP were positively associated with age. Our findings largely correspond with some, but not all other studies. For example, the Mayo Clinic Study of Aging (MCSA) showed higher levels of Aβ42 with older ages (4); but the Health, Aging and Body Composition Study (Health ABC) study found no association between Aβ42 and age (16). MCSA also found increases in Aβ40 levels with age, as did Health ABC (16) and the Systolic Blood Pressure Intervention Trial (SPRINT) trial (5). In the Australian Imaging, Biomarker & Lifestyle Flagship Study of Aging (AIBL) (17), similar to our study, there was no association between baseline Aβ42/Aβ40 and age whereas MCSA showed a negative association between Aβ42/Aβ40 and age. We found no association between pTau181 and baseline age, but the AIBL study found higher pTau181 levels with older ages. Our NfL and GFAP findings were more consistent with findings from the Action to Control Cardiovascular Risk in Diabetes (ACCORD) study (18), AIBL (17), the Austrian Stroke Prevention Family Study (ASPS-Fam) (19), the National Health and Nutrition Examination Survey (NHANES) (20), and SPRINT (5), all showing higher NfL and GFAP levels with increased age. Taken together, these findings suggest that Aβ42, Aβ40, NfL, and GFAP levels increase with age. Relationships between Aβ42/Aβ40, pTau181, and age are less consistent at this time. Of note, one study reported an increase of pTau181 with age only among individuals who were amyloid PET positive (8). Thus, the relationship between pTau181 and age may appear stronger among cohorts who have a higher prevalence of amyloid positivity.

### Gender

In the present study, women had lower levels of Aβ40 than men at baseline. Our results corresponded with the lower Aβ40 levels found in women in the UK Medical Research Council (MRC) National Survey for Health and Development (NSHD) (21). Conversely, there were no gender differences in Aβ40 levels between women and men in the Health ABC cohort (16). While we reported no gender differences in baseline or levels of change over time in Look AHEAD, AIBL (17) similarly showed no differences in Aβ42/Aβ40 by gender; but the MCSA showed lower levels of Aβ42/Aβ40 among women (4). These findings do not paint a clear picture and suggest that more work is needed to understand gender and sex differences in blood-based biomarkers for AD. The various studies we have cited have adjusted for different sets of comorbidities and samples were drawn from different populations with different characteristics which could contribute to different findings.

### Race and Ethnicity

Lower levels of Aβ42 were observed among Black and Hispanic participants compared to White participants. Aβ40 at baseline and change in biomarker levels over the interval followed the same pattern with Black and Hispanic participants having lower levels and less change over time. The opposite trend emerged for the Aβ42/ Aβ40 ratio at baseline and in change over time with White and other/mixed race groups having lower levels and less change over time. Ptau181 levels increased the most over time among White participants compared to other groups. Black participants had the lowest NfL levels on average at baseline. The only biomarker in our study that was not associated with race and ethnicity was GFAP. Others have found similar associations between race and biomarker levels. Hajjar et al. (22), found race differences in biomarkers Aβ42, Aβ40, pTau181, and NfL with African Americans having lower levels than their White counterparts in the Brain, Stress, Hypertension, and Aging Research Program (B-SHARP). In the Health ABC study, investigators similarly found lower levels of Aβ42 and Aβ40 in African American participants compared to White participants (16). Mexican Americans were found to have lower levels of Aβ40, total tau, and Aβ42/Aβ40, in the Health & Aging Brain study among Latino Elders (HABS-HD) (7). However, a study using the NHANES data found no differences in NfL levels by race in models that controlled for age, sex, stroke, diabetes, eGFR, and alcohol consumption (20). More work needs to be done to get a better grasp on these associations as race and ethnicity are social constructs and represent proxies for systemic racism. Associations between biomarkers and race and ethnicity could change depending on the confounders or social determinants of health that are addressed in any given analysis.

### Education

Education levels were associated with GFAP at baseline with those in the lowest education group having the lowest GFAP levels. In the Health ABC study, low education was associated with lower Aβ42 levels (16). It is unclear whether associations between education level and blood-based biomarkers are biologically based or whether higher education is an effective proxy for health advantages due to socio-economic status.

### APOE

APOE ε4 carrier status was only associated with lower Aβ42/Aβ40 ratio at baseline in Look AHEAD. In Health ABC, APOE ε4 carrier status was associated with lower Aβ42 levels (16). In the AIBL study, APOE ε4 carrier status was not associated with biomarker levels after adjustment for age, sex, diagnosis, and amyloid PET Aβ status (17).

### Depressive Symptoms and Antidepressant Usage

Depressive symptoms as measured by the BDI (stratified as BDI<10 vs BDI ≥10), were associated with GFAP levels at baseline. A recent systematic review of GFAP studies indicated associations between GFAP and a number of neurological diseases and disorders, including major depressive disorder as well as Alzheimer’s disease (23). Although there seems to be some association with depression, we were unable to find studies that also investigated antidepressant usage.

### Alcohol and Smoking

Higher alcohol use was associated with higher baseline GFAP levels in our study. In the NHANES study, alcohol use was associated with NfL levels instead, in models that controlled for age, sex, stroke, race, diabetes, and eGFR (20). A history of smoking was associated with an increase in Aβ42 and Aβ40 levels over time in our cohort. We were only able to find one other study that referenced smoking and blood-based AD biomarkers. That study, conducted among patients with heart failure, reported former smokers had lower GFAP levels (24). More work should be done to better understand the interplay between alcohol use, smoking and AD biomarker levels as these are common exposures in older cohorts.

### BMI

Given that Look AHEAD recruited participants with overweight or obesity (mean baseline BMI=34.8±5.3 kg/ m^2^), there was less variation in BMI compared to other studies so we expected to have limited ability to identify differences by BMI. Higher BMI was only significantly associated with lower GFAP levels at baseline, and this association was consistent with a report using BioFINDER data where BMI was inversely correlated with NfL and GFAP (25). In a cohort of 327 community dwelling participants in the ASPS-Fam study, Koini et al. found that BMI was a significant predictor of lower NfL among younger participants (<60) (19). In the AIBL study, lower BMI was correlated with higher pTau181, NfL, and GFAP levels (17).

### Diabetes Duration

More than half of our participants (57%) had diabetes for 5 years or more at baseline. A longer duration of diabetes was associated with higher NfL levels at baseline. Serum NfL levels were also elevated among diabetics in an HNANES analysis compared to those without diabetes (20). Using data from the HABS-HD study, O’Bryant et al. found relationships between the presence of diabetes (not duration) and higher levels of Aβ42, Aβ40, and NfL (7). The NHANES study examined the association between NfL and the presence of diabetes (also not considering duration), and found that diabetes was associated with higher NfL levels in models that controlled for age, sex, stroke, race, diabetes, eGFR, and alcohol consumption (20).

### HbA1c

HbA1c levels <7% were associated with higher Aβ42 and Aβ40 at baseline and lower levels of increase from baseline to end of intervention. HbA1c levels ≥7% were associated with greater NfL increases from baseline. In the ACCORD Study, NfL was similarly positively associated with HbA1c (18). In the HABS-HD study, higher HbA1c was also related to higher Aβ42, Aβ40, and NfL (7).

### Diabetes Treatment

Participants not using diabetes medications had lower Aβ42/Aβ40 and NfL levels at baseline. While few studies have reported specifically on the relationship between diabetes treatment and blood-based biomarkers for AD, several studies have investigated the associations between diabetes treatment and cognitive outcomes (26). Future work should examine more closely the relationship between specific diabetes medication classes and blood-based biomarkers as they have different effects (27).

### Peripheral Neuropathy

We found no significant relationships between any of the biomarkers and neuropathy. In contrast, a meta-analysis of 36 studies found significant associations between neuropathy and higher NfL levels (28). It is possible our data failed to reflect this association because the generally higher BMI levels may dilute NfL levels in our cohort.

### e GFR

Estimated glomerular filtration rate (eGFR) has been consistently associated with biomarker levels in the literature (8). A higher filtration rate (i.e., higher eGFR) indicates better kidney function and tends to be associated with lower levels of biomarkers in the blood. In Look AHEAD, higher eGFR was associated with lower baseline levels of Aβ42, Aβ40, Aβ42/Aβ40 ratio, NfL, and GFAP. In the ASPS-Fam study, ACCORD, and NHANES, renal function measured by eGFR was similarly associated with NfL levels (18-20). A higher eGFR was also significantly related to lower Aβ42, Aβ40, Tau, and NfL; and higher levels of Aβ42/Aβ40 in the HABS-HD study (7). In SPRINT, after adjustment for age, both Aβ40, and NfL were negatively associated with eGFR. Of all the factors studied here, eGFR seems to have consistent associations with biomarkers across studies similar to increased age.

### Blood Pressure

Self-reported hypertension was associated with higher baseline Aβ42 and Aβ40 and greater decline in the Aβ42/Aβ40 ratio from baseline to end of intervention in our study. In the HABS-HD and ACCORD studies, hypertension was related to NfL (7, 18). In SPRINT, greater increases in NfL were seen in the intensive treatment (blood pressure lowering) arm of the study, however the association was attenuated when adjusted for eGFR (5).

### Dyslipidemia

We examined potential associations with self-reported high cholesterol and found no significant associations. In the HABS-HD Study, dyslipidemia was related to Aβ42, Aβ40, and NfL; and higher levels of Aβ42/Aβ40 in models that were adjusted for age, sex, and education (7).

### History of CVD

A history of CVD at baseline in Look AHEAD, defined as self-reported myocardial infarction, heart bypass surgery, coronary artery bypass graft, carotid endarterectomy, lower leg angioplasty, aortic aneurysm, congestive heart failure and stroke, was associated with a greater increase in Aβ42 and Aβ40 over time. History of CVD was also associated with higher baseline GFAP levels. Syrjanan et al. examined history of stroke and myocardial infarction and found that stroke was related to higher baseline NfL and total tau, while myocardial infarction was only related to total tau (4).

There are several key findings from this exercise. First, blood-based biomarker levels at baseline in our cohort were associated with 15 out of 18 variables, many of which represent conditions that are very common in older adults. Interestingly, in this study of participants with diabetes and overweight or obesity, diabetes treatment was associated with Aβ42, Aβ40, Aβ42/Aβ40, and NfL, while BMI was associated only with GFAP. As we have outlined, these associations are in general agreement with the limited number of studies that have evaluated these biomarkers in other cohorts (4, 5, 7, 16-22, 25, 29, 30). For example, when considering our work and others, we observe general agreement that Aβ42, Aβ40, NfL, and GFAP levels go up with age. There is a strong suggestion that levels of Aβ42, Aβ40, pTau181 are lower among persons with self-reported Black and Hispanic race and ethnicity. Second, biomarker levels were not associated with legacy effects of our ILI intervention. Third, changes in the biomarker levels over time are associated with race (Aβ42, Aβ40, Aβ42/Aβ40, and NfL), smoking (Aβ42, Aβ40), HbA1c levels (Aβ42, Aβ40, pTau181), and history of CVD (Aβ42, Aβ40). Evaluation of variables that are associated with change in biomarker levels over time suggests potential for malleability. Whether this translates into modifiable lifestyle behaviors is currently unknown.

There has been much progress in the technology to evaluate blood-based biomarkers for neurodegenerative disease. As blood samples are more distal than CSF, the assays for blood samples have to be particularly sensitive and are subject to additional complexities inherent in their measurement in blood (31). As the field begins to use these markers as screening tools for trials or as clinical indicators of brain health, it is critically important to understand all the patient/participant characteristics that can influence their measurement. In our study, all the participants have T2D and all of them had overweight or obesity at baseline, both of which are characteristics associated with increased risk of cognitive impairment and dementia in later life.

### Limitations

This study is not without limitations. There was no baseline cognitive assessment as it was not a primary focus of the original Look AHEAD clinical trial. Therefore, participants were not excluded based on the presence of cognitive impairment at baseline. Cognitive impairment was not assessed until after the end of the intervention. Nonetheless, our rigorous screening procedures, designed to ascertain whether participants could execute the protocol, would have effectively excluded those with clear impairment. Our self-reported designations of race and ethnicity were not collected in a mutually exclusive fashion, thereby conflating the two. Randomization facilitated comparable demographic and health characteristics across study arms at baseline, and there is no reason to suspect that the two groups would have differed in cognitive performance at baseline either.

There may be other age-related chronic diseases that influence plasma levels of biomarkers for which we did not account. Our findings are perhaps only generalizable to a high-risk subset of the population, i.e., older adults with T2D and overweight/obesity. However, this group represents a growing at-risk portion of the population. While we tried to put our results in context with other studies, we acknowledge that there are differences in study designs including the methods used to ascertain biomarker level, the proportions of people of different races and ethnicities included, and assumptions about cognitive status at baseline. We also acknowledge that while we are looking at differences among important clinical subgroups, we are unable to determine the degree to which these reflect underlying differences in neuropathology. Finally, a strength of the work is the fact that Look AHEAD was a randomized controlled clinical trial, conducted using rigorous methods.

## Conclusions

Blood-based biomarkers for neurodegenerative disorders have great potential for identifying individuals at risk for cognitive decline and dementia, however more work needs to be done in this area before they can be used clinically. Much of the work to date in biomarker studies has focused on clinical samples, comparing participants with and without clinically diagnosed dementing illness, often with confirmatory and Aβ and Tau PET imaging. Emerging work similar to results we present suggests that a number of participant or patient characteristics need to be considered when interpreting blood-based biomarkers. Our study of biomarkers in the Look AHEAD cohort reveals a number of participant characteristics that correlate with biomarker levels in a sample of individuals with diabetes and overweight or obesity, which should be considered when developing clinical applications for these biomarkers.

## Supplemental Materials

Supplemental Tables Supplementary PDF file supplied by authors.

**Supplemental Table 1.** Baseline Characteristics of Participants by Inclusion Status

Characteristic	Included (n= 779)	Excluded (n=4366)	*p*-value
Randomized Group, No. (%)			0.593
Diabetes Support and Education	383 (49.2%)	2192 (50.2%)	
Intensive Lifestyle Intervention	396 (50.8%)	2174 (49.8%)	
Age, mean ± SD, years	61.4 ± 6.2	58.2 ± 6.8	<.0001
Age Category (years), No. (%)			<.0001
45 - 55	122 (15.7%)	1498 (34.3%)	
56 - 65	484 (62.1%)	2167 (49.6%)	
66 - 76	173 (22.2%)	701 (16.1%)	
Gender, No. (%)			0.041
Men	341 (43.8%)	1741 (39.9%)	
Women	438 (56.2%)	2625 (60.1%)	
Race, No. (%)			0.002
African American / Black (not Hispanic)	130 (16.7%)	674 (15.4%)	
American Indian / Native American / Alaskan Native	16 ( 2.1%)	242 ( 5.5%)	
Asian/Pacific Islander	6 ( 0.8%)	44 ( 1.0%)	
White	516 (66.2%)	2736 (62.7%)	
Hispanic	97 (12.5%)	583 (13.4%)	
Other/Mixed	14 ( 1.8%)	86 ( 2.0%)	
Years of Education, No. (%)			<.0001
< 13 years	199 (25.5%)	821 (18.8%)	
13 - 16 years	269 (34.5%)	1647 (37.7%)	
> 16 years	288 (37.0%)	1806 (41.4%)	
Missing	23 (3.0%)	92 (2.1)	
*APOE* ε4 carrier status, No. (%)	151 (22.8%)	818 (23.6%)	0.660
Beck Depression Inventory ≥10, No. (%)	129 (16.7%)	795 (18.3%)	0.283
Beck Depression Inventory ≥24, No. (%)	5 ( 0.6%)	31 ( 0.7%)	0.840
Antidepressant Medication, No. (%)	115 (15.1%)	734 (17.4%)	0.117
Baseline Alcohol Consumption, No. (%)			0.612
None	523 (67.1%)	2969 (68.0%)	
< 21 oz/wk	154 (19.8%)	880 (20.2%)	
≥ 21 oz/wk	102 (13.1%)	517 (11.8%)	
Baseline Smoking, No. (%)			0.065
Never	397 (51.2%)	2179 (50.0%)	
Past	357 (46.0%)	1974 (45.3%)	
Present	22 ( 2.8%)	205 ( 4.7%)	
Body Mass Index, mean ± SD, kg/m^2^	34.8 ± 5.3	36.1 ± 6.0	<.0001
Body Mass Index, No. (%)			<.0001
< 30 kg/m^2^	149 (19.1%)	616 (14.1%)	
30-39 kg/m^2^	509 (65.3%)	2722 (62.3%)	
40+ kg/m^2^	121 (15.5%)	1028 (23.5%)	
Diabetes Duration (≥5years), No. (%)	441 (57.2%)	2327 (53.7%)	0.072
HbA1c %, mean ± SD	7.2 ± 1.1	7.3 ± 1.2	0.063
HbA1c, No. (%)			0.153
< 7%	366 (47.0%)	1985 (45.5%)	
7-9%	355 (45.6%)	1961 (44.9%)	
≥ 9%	58 ( 7.4%)	420 ( 9.6%)	
Baseline Diabetes Treatment, No. (%)			0.802
No Meds	105 (13.6%)	566 (13.1%)	
Oral Meds, No insulin	553 (71.5%)	3075 (71.2%)	
Oral Meds + Insulin	115 (14.9%)	680 (15.7%)	
Baseline Insulin Use, No. (%)	115 (15.1%)	680 (16.2%)	0.450
Baseline Peripheral Neuropathy, No. (%)	123 (15.8%)	777 (17.8%)	0.174
GFR <=60	29 ( 4.0%)	173 ( 4.0%)	0.046
GFR 60-90	292 (40.6%)	1563 (35.9%)	
GFR>=90	398 (55.4%)	2620 (60.1%)	
Baseline Hypertension, No. (%)	651 (83.6%)	3630 (83.1%)	0.769
Baseline Dyslipidemia, No. (%)	697 (89.5%)	3806 (87.2%)	0.074
Baseline CVD History, No. (%)	130 (16.7%)	582 (13.3%)	0.012

Abbreviations: Aβ, Amyloid beta; *APOE* ε4, Apolipoprotein E gene, ε4 carrier status; CVD, cardiovascular disease; DL, deciliter; DSE, diabetes support and education; GFAP, glial fibrillary acidic protein; GFR, glomerular filtration rate; HbA1c, hemoglobin A1c; Hx, history; kg/m^2^, ILI, intensive lifestyle intervention; kilogram per square meter; NfL, neurofilament light chain; OM, oral medication; oz, ounces; SD, standard deviation, Trt, treatment; wk, week; w/, with; w/out, without; Yrs, years

**Supplemental Table 2.** Summary of selected characteristics of cited studies.

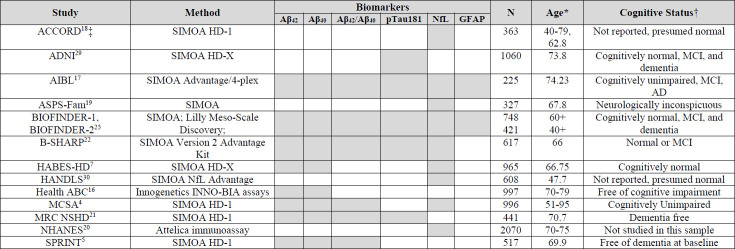

Shaded boxes indicate which biomarkers were measured in the study. *Ages indicated are means, medians, and ranges per the individual determined at baseline or initial biomarker measurement. ‡ACCORD sample includes only participants with diabetes. ACCORD: Action to Control Cardiovascular Risk in Diabetes ADNI: Alzheimer’s Disease Neuroimaging Initiative AIBL: Australian Imaging, Biomarker & Lifestyle Flagship Study of Aging ASPS-Fam: Austrian Stroke Prevention Family Study BioFINDER-1: Biomarkers For Identifying Neurodegenerative Disorders BioFINDER-2: Early and Reliably Health and Development B-SHARP: Brain, Stress, Hypertension, and Aging Research Program HABS-HD: Health & Aging Brain study among Latino HANDLES: Healthy Aging in Neighborhoods of Diversity Health ABC: Health, Aging and Body Composition MCSA: Mayo Clinic Study of Aging MRC NSHD: Medical Research Council National NHANES: National Health and Nutrition Examination SPRINT: Systolic Blood Pressure Intervention Trial

## Clinical Sites

The Johns Hopkins University: Jeanne M. Clark, MD, MPH1; Lee Swartz2; Dawn Jiggetts2; Jeanne Charleston, RN3; Lawrence Cheskin, MD3; Nisa M. Maruthur, MD, MHS 3; Scott J. Pilla, MD, MHS3; Danielle Diggins; Mia Johnson. Pennington Biomedical Research Center George: A. Bray, MD1; Frank L. Greenway, MD1; Donna H. Ryan, MD3; Catherine Champagne, PhD, RD3; Valerie Myers, PhD3; Jeffrey Keller, PhD3; Tiffany Stewart, PhD3; Jennifer Arceneaux, RN2; Karen Boley, RD, LDN2; Greta Fry, LPN; Lisa Jones; Kim Landry; Melissa Lingle; Marisa Smith2. The University of Alabama at Birmingham: Cora E. Lewis, MD, MSPH1; Sheikilya Thomas, PhD, MPH2; Stephen Glasser, MD3; Gareth Dutton, PhD3; Amy Dobelstein; Sara Hannum; Anne Hubbell, MS; DeLavallade Lee; Phyllis Millhouse, L. Christie Oden; Cathy Roche, PhD, RN, BSN; Jackie Grant; Janet Turman. Harvard Center: Massachusetts General Hospital. David M. Nathan, MD1; Valerie Goldman, MS, RDN2; Linda Delahanty, MS, RDN3; Mary Larkin, MS, RN; Kristen Dalton, BS; Roshni Singh, BS; Melanie Ruazol, BS. Joslin Diabetes Center: Joslin Diabetes Center: Medha N Munshi, MD1; Sharon D. Jackson, CCRC, MS, RD, CDE2; Roeland J.W. Middelbeek MD3; A. Enrique Caballero, MD, Anthony Rodriguez. Beth Israel Deaconess Medical Center: George Blackburn, MD, PhD1*; Christos Mantzoros, MD, DSc3; Ann McNamara, RN. University of Colorado Anschutz Medical Campus: Holly Wyatt, MD1; James O. Hill, PhD1; Jeanne Anne Breen, MS2; Marsha Miller, MS, RD2; Debbie Bochert; Suzette Bossart; Paulette Cohrs, RN, BSN; Susan Green; April Hamilton, BS, CCRC; Eugene Leshchinskiy; Loretta Rome, TRS. The University of Tennessee Health Science Center: University of Tennessee East. Karen C. Johnson, MD, MPH1; Beate Griffin, RN, BS2; Mace Coday, PhD3; Donna Valenski, Linda Jones; Karen Johnson, RN. University of Tennessee Downtown: Karen C. Johnson, MD, MPH1; Beate Griffin, RN, BS2; Helmut Steinburg, MD3. University of Minnesota: Robert W. Jeffery, PhD1; Tricia Skarphol, MA2; John P. Bantle, MD3; J. Bruce Redmon, MD3; Kerrin Brelje, MPH, RD; Carolyne Campbell; Mary Ann Forseth, BA; Soni Uccellini, BS; Mary Susan Voeller, BA. Columbia University Medical Center: Blandine Laferrère, MD, PhD1; Xavier Pi-Sunyer, MD1; Jennifer Patricio, MS2; Jose Luchsinger, MD1; Priya Palta, PhD,MHS3; Jennifer Patricio, MS2; Sarah Lyon, Kim Kelly. University of Pennsylvania: Thomas A. Wadden, PhD1; Barbara J. Maschak-Carey, MSN, CDE2; Robert I. Berkowitz, MD3; Ariana Chao, PhD, CRNP3; Renee Davenport; Katherine Gruber, CRNP; Sharon Leonard, RD; Olivia Walsh, BA. University of Pittsburgh: John M. Jakicic, PhD1; Jacqueline Wesche-Thobaben, RN, BSN, CDE2; Lin Ewing, PhD, RN3; Andrea Hergenroeder, PhD, PT, CCS3; Mary Korytkowski, MD3; Susan Copelli, BS, CTR; Rebecca Danchenko, BS; Diane Ives, MPH; Juliet Mancino, MS, RD, CDE, LDN; Lisa Martich, BS, RD, LDN; Meghan McGuire, MS; Tracey Y. Murray, BS; Linda Semler, MS, RD, LDN; Kathy Williams, RN, MHA. The Miriam Hospital/Brown Medical School: Rena R. Wing, PhD1; Caitlin Egan, MS2; Elissa Jelalian, PhD3; Jeanne McCaffery, PhD3 ; Kathryn Demos McDermott, PhD3; Jessica Unick, PhD3; Kirsten Annis, BA; Jose DaCruz; Ariana Rafanelli, BA. The University of Texas Health Science Center at San Antonio: Helen P. Hazuda, PhD1; Juan Carlos Isaac, CCRC, BSN2; Prepedigna Hernandez, RN. VA Puget Sound Health Care System / University of Washington: Steven E. Kahn, MB, ChB1; Edward J. Boyko, MD, MPH3; Elaine Tsai, MD3; Lorena Wright, MD3; Karen Atkinson, RN, BSN2; Ivy Morgan-Taggart; Jolanta Socha, BS; Heidi Urquhart, RN. Southwestern American Indian Center, Phoenix, Arizona and Shiprock, New Mexico: William C. Knowler, MD, DrPH1; Paula Bolin, RN, MC2; Harelda Anderson, LMSW2, Sara Michaels, MD3; Ruby Johnson; Patricia Poorthunder; Janelia Smiley. University of Southern California: Anne L. Peters, MD1; Siran Ghazarian, MD2; Elizabeth Beale, MD3; Edgar Ramirez; Gabriela Rodriguez, MA; Valerie Ruelas MSW, LCSW; Sara Serafin-Dokhan; Martha Walker, RD; Marina Perez.

## Coordinating Center

Wake Forest University Mark A. Espeland, PhD1; Kathleen Hayden, PhD1; Judy L. Bahnson, BA, CCRP3; Lynne E. Wagenknecht, DrPH3; David Reboussin, PhD3; Nicholas Pajewski, PhD3; Jingzhong Ding, PhD3; Gagan Deep, PhD3; Stephen R. Rapp, PhD3; Bonnie C. Sachs, PhD3; Jerry M. Barnes, MA; Tara D. Beckner; Delilah R. Cook; Joni Evans, MS; Katie Garcia, MS; Sarah A. Gaussoin, MS; Carol Kittel, MS; Lea Harvin, BS; Marjorie Howard, MS; Rebecca H. Neiberg, MS; Jennifer Walker, MS; Michael P. Walkup, MS
