# Supplementary material for: Association between Modifiable Risk Factors and Levels of Blood-Based Biomarkers of Alzheimer’s and Related Dementias in the Look AHEAD Cohort
Source: JAR Life. 2024 Jan 5;13:1–21. doi: 10.14283/jarlife.2024.1 (PMC10775955; doi:10.14283/jarlife.2024.1)
Supplement: Supplemental Tables — Supplementary PDF file supplied by authors. [file jarlife-13-0001-S1.pdf]

Supplemental Tables

Supplemental Table 1. Baseline Characteristics of Participants by Inclusion Status

Characteristic	Included (n= 779)	Excluded (n=4366)	*p*-value
Randomized Group, No. (%)			0.593
Diabetes Support and Education	383 (49.2%)	2192 (50.2%)	
Intensive Lifestyle Intervention	396 (50.8%)	2174 (49.8%)	
Age, mean ± SD, years	61.4 ± 6.2	58.2 ± 6.8	<.0001
Age Category (years), No. (%)			<.0001
45 - 55	122 (15.7%)	1498 (34.3%)	
56 - 65	484 (62.1%)	2167 (49.6%)	
66 - 76	173 (22.2%)	701 (16.1%)	
Gender, No. (%)			0.041
Men	341 (43.8%)	1741 (39.9%)	
Women	438 (56.2%)	2625 (60.1%)	
Race, No. (%)			0.002
African American / Black (not Hispanic)	130 (16.7%)	674 (15.4%)	
American Indian / Native American / Alaskan Native	16 (2.1%)	242 (5.5%)	
Asian/Pacific Islander	6 (0.8%)	44 (1.0%)	
White	516 (66.2%)	2736 (62.7%)	
Hispanic	97 (12.5%)	583 (13.4%)	
Other/Mixed	14 (1.8%)	86 (2.0%)	
Years of Education, No. (%)			<.0001
< 13 years	199 (25.5%)	821 (18.8%)	
13 - 16 years	269 (34.5%)	1647 (37.7%)	
> 16 years	288 (37.0%)	1806 (41.4%)	
Missing	23 (3.0%)	92 (2.1)	
APOE ε4 carrier status, No. (%)	151 (22.8%)	818 (23.6%)	0.660
Beck Depression Inventory ≥10, No. (%)	129 (16.7%)	795 (18.3%)	0.283
Beck Depression Inventory ≥24, No. (%)	5 (0.6%)	31 (0.7%)	0.840
Antidepressant Medication, No. (%)	115 (15.1%)	734 (17.4%)	0.117
Baseline Alcohol Consumption, No. (%)			0.612
None	523 (67.1%)	2969 (68.0%)	

Characteristic	Included (n= 779)	Excluded (n=4366)	*p*-value
< 21 oz/wk	154 (19.8%)	880 (20.2%)	
≥ 21 oz/wk	102 (13.1%)	517 (11.8%)	
Baseline Smoking, No. (%)			0.065
Never	397 (51.2%)	2179 (50.0%)	
Past	357 (46.0%)	1974 (45.3%)	
Present	22 (2.8%)	205 (4.7%)	
Body Mass Index, mean ± SD, kg/m²	34.8 ± 5.3	36.1 ± 6.0	<.0001
Body Mass Index, No. (%)			<.0001
< 30 kg/m²	149 (19.1%)	616 (14.1%)	
30-39 kg/m²	509 (65.3%)	2722 (62.3%)	
40+ kg/m²	121 (15.5%)	1028 (23.5%)	
Diabetes Duration (≥5years), No. (%)	441 (57.2%)	2327 (53.7%)	0.072
HbA1c %, mean ± SD	7.2 ± 1.1	7.3 ± 1.2	0.063
HbA1c, No. (%)			0.153
< 7%	366 (47.0%)	1985 (45.5%)	
7-9%	355 (45.6%)	1961 (44.9%)	
≥ 9%	58 (7.4%)	420 (9.6%)	
Baseline Diabetes Treatment, No. (%)			0.802
No Meds	105 (13.6%)	566 (13.1%)	
Oral Meds, No insulin	553 (71.5%)	3075 (71.2%)	
Oral Meds + Insulin	115 (14.9%)	680 (15.7%)	
Baseline Insulin Use, No. (%)	115 (15.1%)	680 (16.2%)	0.450
Baseline Peripheral Neuropathy, No. (%)	123 (15.8%)	777 (17.8%)	0.174
GFR <=60	29 (4.0%)	173 (4.0%)	0.046
GFR 60-90	292 (40.6%)	1563 (35.9%)	
GFR>=90	398 (55.4%)	2620 (60.1%)	
Baseline Hypertension, No. (%)	651 (83.6%)	3630 (83.1%)	0.769
Baseline Dyslipidemia, No. (%)	697 (89.5%)	3806 (87.2%)	0.074
Baseline CVD History, No. (%)	130 (16.7%)	582 (13.3%)	0.012

Abbreviations: Aβ, Amyloid beta; *APOE* ε4, Apolipoprotein E gene, ε4 carrier status; CVD, cardiovascular disease; DL, deciliter; DSE, diabetes support and education; GFAP, glial fibrillary acidic protein; GFR, glomerular filtration rate; HbA1c, hemoglobin A1c; Hx, history;

kg/m^2, ILI, intensive lifestyle intervention; kilogram per square meter; NfL, neurofilament light chain; OM, oral medication; oz, ounces; SD, standard deviation, Trt, treatment; wk, week; w/, with; w/out, without; Yrs, years

Supplemental Table 2. Summary of selected characteristics of cited studies.

Study	Method	Biomarkers						N	Age*	Cognitive Status†
		$A\beta_{42}$	$A\beta_{40}$	$A\beta_{42}/A\beta_{40}$	pTau181	NfL	GFAP			
ACCORD[18]‡	SIMOA HD-1					■		363	40-79, 62.8	Not reported, presumed normal
ADNI[29]	SIMOA HD-X				■			1060	73.8	Cognitively normal, MCI, and dementia
AIBL[17]	SIMOA Advantage/4-plex	■	■	■	■	■	■	225	74.23	Cognitively unimpaired, MCI, AD
ASPS-Fam[19]	SIMOA					■		327	67.8	Neurologically inconspicuous
BIOFINDER-1, BIOFINDER-2[25]	SIMOA; Lilly Meso-Scale Discovery;	■	■	■	■	■	■	748<br>421	60+<br>40+	Cognitively normal, MCI, and dementia
B-SHARP[22]	SIMOA Version 2 Advantage Kit	■	■	■	■	■		617	66	Normal or MCI
HABES-HD[7]	SIMOA HD-X	■	■			■		965	66.75	Cognitively normal
HANDLS[30]	SIMOA NfL Advantage					■		608	47.7	Not reported, presumed normal
Health ABC[16]	Innogenetics INNO-BIA assays	■	■					997	70-79	Free of cognitive impairment
MCSA[4]	SIMOA HD-1	■	■	■		■		996	51-95	Cognitively Unimpaired
MRC NSHD[21]	SIMOA HD-1	■	■	■	■			441	70.7	Dementia free
NHANES[20]	Attelica immunoassay					■		2070	70-75	Not studied in this sample
SPRINT[5]	SIMOA HD-1	■	■	■				517	69.9	Free of dementia at baseline

Shaded boxes indicate which biomarkers were measured in the study. *Ages indicated are means, medians, and ranges per the individual studies. †Status reported in papers, determined at baseline or initial biomarker measurement. ‡ACCORD sample includes only participants with diabetes.

ACCORD: Action to Control Cardiovascular Risk in Diabetes
ADNI: Alzheimer's Disease Neuroimaging Initiative
AIBL: Australian Imaging, Biomarker & Lifestyle Flagship Study of Aging
ASPS-Fam: Austrian Stroke Prevention Family Study
BioFINDER-1: Biomarkers For Identifying Neurodegenerative Disorders
BioFINDER-2: Early and Reliably
B-SHARP: Brain, Stress, Hypertension, and Aging Research Program
HABS-HD: Health & Aging Brain study among Latino Elders
HANDLES: Healthy Aging in Neighborhoods of Diversity across the Life Span
Health ABC: Health, Aging and Body Composition Study
MCSA: Mayo Clinic Study of Aging
MRC NSHD: Medical Research Council National Survey for Health and Development
NHANES: National Health and Nutrition Examination Survey
SPRINT: Systolic Blood Pressure Intervention Trial
